# 26.4 LANGUAGE DISTURBANCE AS A PREDICTOR OF PSYCHOSIS ONSET IN YOUTH AT ENHANCED CLINICAL RISK

**DOI:** 10.1093/schbul/sby014.109

**Published:** 2018-04-01

**Authors:** Cheryl Corcoran, Facundo Carrillo, Diego Fernández Slezak, Casimir Klim, Gillinder Bedi, Daniel Javitt, Carrie Bearden, Guillermo Cecchi

**Affiliations:** 1 Icahn School of Medicine at Mount Sinai; 2 Universidad de Buenos Aires; 3 University of Michigan Medical School; 4 NYSPI, Columbia University; 5 Columbia University; 6 University of California, Los Angeles; 7 IBM TJ Watson Research Center

## Abstract

**Background:**

Language offers a privileged view into the mind; it is the basis by which we infer others’ thoughts. Subtle language disturbance is evident in schizophrenia prior to psychosis onset, including decreases in coherence and complexity, as measured using clinical ratings in familial and clinical high-risk (CHR) cohorts. Bearden et al previously used manual linguistic analysis of baseline speech transcripts in CHR to show that illogical and referential thinking, and poverty of content, predict later psychosis onset. Then, Bedi et al used automated natural language processing (NLP) of CHR transcripts to show that decreased semantic coherence and reduction in syntactic complexity predicted psychosis onset. To determine validity and reproducibility, we have applied automated NLP methods, with machine learning, to Bearden’s original CHR transcripts to identify a language profile predictive of psychosis.

**Methods:**

Participants in the Bearden UCLA cohort include 59 CHR, of whom 19 developed psychosis (CHR+) within 2 years, whereas 40 did not (CHR-), as well as 16 recent-onset psychosis and 21 healthy individuals, similar in demographics; speech was elicited using Caplan’s “Story Game. Participants in the Bedi NYC cohort include 34 CHR (29 CHR+), with speech elicited using open-ended interview. Speech was audiotaped, transcribed, de-identified and then subjected to latent semantic analysis to determine coherence and part-of-speech tagging to characterize syntactic structure and complexity. A machine-learning speech classifier of psychosis onset was derived from the UCLA CHR cohort, and then applied both to the NYC CHR cohort and to the UCLA psychosis/control comparison, with convex hull (three-dimension depiction of model) and receiver operating characteristics analyses. Correlational analyses with demographics, symptoms and manual linguistic features were also done.

**Results:**

A four-factor model language classifier derived from the UCLA CHR cohort that comprised three semantic coherence variables and one syntax (usage of possessive pronouns) predicted psychosis t with accuracy of 83% (intra-protocol) for UCLA CHR, 79% (cross-protocol) for NYC CHR, and 72% for discriminating psychosis from normal speech (UCLA psychosis/control). Convex hulls were defined as the smallest space containing all datapoints within a set for CHR- or healthy controls: these convex hulls showed substantial overlap, with CHR+ and psychosis speech datapoints largely outside these convex hulls. Coherence was associated with age, but speech variables did not vary by gender, race, or socioeconomic status in this study. While automated text features were unrelated to prodromal symptom severity, they were highly correlated with manual text features (r = 0.7, p < .000001).

**Discussion:**

In this small preliminary study, we identified and cross-validated a robust language classifier of psychosis risk that comprised measures of semantic coherence (flow of meaning in language) and syntactic usage (usage of possessive pronouns). This classifier had utility in discriminating speech in individuals with recent-onset psychosis from the norm. It demonstrated concurrent validity in that it was highly correlated with manual linguistic features previously identified by Bearden et al, important as automated methods are fast and inexpensive. Automated language features were unrelated to sex, ethnicity or social class in these small samples, and semantic coherence increased with age, consistent with prior studies of normal language development. Of interest, overlapping convex hulls could be defined for groups of individuals without psychosis (UCLA CHR-, NYC CHR- and UCLA healthy), suggesting a constrained hull of normal language in respect to syntax and semantics, from which pre-psychosis and psychosis speech deviates. The RDoC linguistic corpus-based variables of semantic coherence and syntactic structure hold promise as biomarkers of psychosis risk and expression, with initial validation and reproducibility. Next steps in biomarker development include larger multisite studies with standardization of protocols for speech elicitation, test-retest, and attention to traction/feasibility, acceptability, cost, and utility. Mechanistic studies can also yield neural and physiological correlates of abnormal semantic coherence and syntax.

